# Cultivation strategies of a BA/F3 cell line for fundamental cell research

**DOI:** 10.1186/1753-6561-5-S8-P48

**Published:** 2011-11-22

**Authors:** Martin Schaletzky, Oscar Platas Barradas, Henning Sievert, Stefan Balabanov, An-Ping Zeng, Ralf Pörtner

**Affiliations:** 1 Institute of Bioprocess and Biosystems Engineering, Hamburg University of Technology, Hamburg, D-21073, Germany; 2 Cancer Center Hamburg, University Medical Center Hamburg-Eppendorf (UKE), Hamburg, D-20246, Germany

## Background

During the chronic myelogenous leukemia (CML) the Bcr-Abl oncoprotein is produced, which leads to unregulated cell proliferation. CML is treated with one of several targeting therapies such as imatinib, (formerly STI-571 [Glivec; Novartis, Switzerland]) a selective inhibitor that blocks tyrosin kinase activity of the Bcr-Abl oncoprotein. Apart from the second generation Bcr-Abl inhibitors, identifying novel direct or indirect downstream targets of Bcr-Abl could contribute significantly to the development of new synergistic treatment strategies against CML. The effects of imatinib on the protein expression of Bcr-Abl positive cells are being investigated [1]. A protein which is downregulated during treatment with imatinib (eukaryotic translation initiation factor eIF5A) was identified. This protein is a potentially promising target for single-agent and combined-treatment strategies for CML. For protein complex identification a high cell number is needed. This is difficult to be obtained reproducibly with flask cultures or roller bottles. The aim of this project was to develop and establish a reproducible bioreactor cultivation of murine suspension cell lines (BA/F3 p210), which yields a total cell number close to 1·10^10^ cells required for analytics. Cells should be in exponential growth under constant culture conditions at the time of harvest. A small stirred tank bioreactor with a working volume of 150 mL was used to study and compare different operation modes: *batch*, *fed-batch* and continuous. Cell growth and glucose consumption were assessed as main culture parameters.

## Material and methods

**Cell lines:** BA/F3 p210 and BA/F3 p210 eIF5A-2 (BA/F3 = mouse pro B cells, p210 = Bcr-Abl oncoprotein (210kDa), eIF5A-2 = isoform of the eukaryotic translation initiation factor eIF5A).

In a first step, a working cell bank was established and cell growth was characterized in T-flasks. Afterwards, different cultivation modes were tested in a stirred tank bioreactor (Vario1000, Medorex, Germany) as follows:

***batch:*** Cultivation volume V_start_ = 350 mL, duration: 40 h

***fed-batch:*** Cultivation volume V_start_ = 345 mL, duration: 64 h, Feeding took place every time Glucose concentration fell below 2 mM. Feed medium consisted on a mixture of batch medium and higher concentrations of glucose and glutamine.

**Continuous:** Cultivation volume V_start_ = 115 mL, dilution rate D = 0.049 h^-1^ duration: 118.5 h.

The scale-up experiment was performed in a 5 L stirred bench-top bioreactor (Biostat B, Sartorius Stedim Biotech GmbH) with pH and DO control.

## Results and conclusions

In batch mode, the maximum viable cell density during exponential growth was VCD_max_ = 14.7·10^5^ cells mL^-1^. In fed-batch mode VCD_max_ = 22.6·10^5^ cells mL^-1^. This higher cell density is an advantage over the batch culture mode. It was not possible to obtain higher cell densities in this mode, since the feed medium consisted on a formulation for batch culture with further addition of glucose and glutamine. In continuous mode the highest possible cell density was maintained in the bioreactor, in order to produce continuously cells for further treatment. A maximum cell yield of 8.3·10^6^ cells h^-1^ could be harvested from the bioreactor. After scale-up, this yield might be increased, so that the needed cell number could be harvested in only few days. A disadvantage of the continuous process with cell harvest was observed for the storage process, since cell lysis took place after storage at 4 °C.

**Figure 1:**
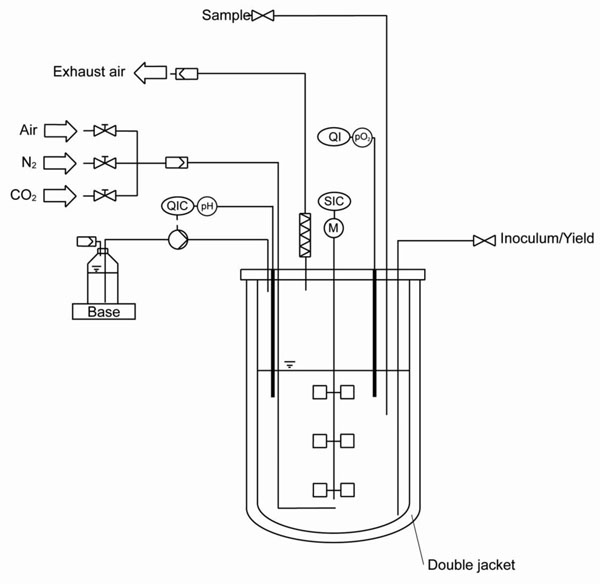
Schematic diagram of the final *batch* process in a 5 L bioreactor that yields a total cell number close to 1·10^10^

A first approach for scale-up was performed in the 5 L bioreactor (Figure 1), where the maximum cell density during exponential phase allowed for the needed cell number. Regarding the required reproducibility for cultivation, the 5 L batch mode was preferred over T-flasks due to the possibility for control of process variables like pH and pO_2_.

**Figure 2:**
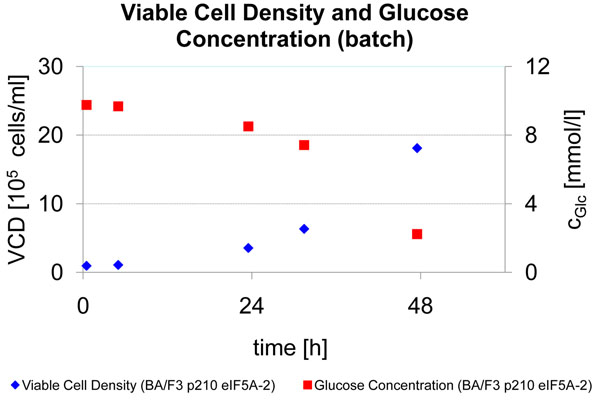
V_start_ = 5090 mL, max. viable cell density in exponential growth after 39.5 hours VCD_max_ = 18.1·10^5^ cells mL^-^

Compared to T-flasks, glucose uptake during bioreactor cultivation was much higher, which led to lower final-cell-density yields. fed-batch and continuous modes were firstly favored due the theoretical final cell numbers reached during culture. However, the difference in growth, limitation of bioreactor volume and the need of a special medium formulation for higher cell densities during fed-batch, limited the final yield. Continuous mode with temperature reduction of harvested cells allowed for constant cell production in exponential phase. On the other hand, storage of intact cells was limited probably due to protease action. The 150 mL batch cultivation was scaled up to 5 L in a stirred bench-top bioreactor (Biostat B, Sartorius Stedim Biotech GmbH).
